# Complex Pathology and Management in the Obstetric Patient: A Narrative Review for the Anesthesiologist

**DOI:** 10.7759/cureus.17196

**Published:** 2021-08-15

**Authors:** Lia Metzger, Menachem Teitelbaum, Garret Weber, Sangeeta Kumaraswami

**Affiliations:** 1 Anesthesiology, Beth Israel Deaconess Medical Center, Harvard Medical School, Boston, USA; 2 Medicine, New York University, Mineola, USA; 3 Anesthesiology, New York Medical College, Valhalla, USA

**Keywords:** obstetric, consultation, preanesthetic, cardiovascular, respiratory, gastrointestinal, psychiatric, substance abuse, history, physical examination

## Abstract

Pregnant patients may present with multiple complex comorbidities that can affect peripartum management and anesthetic care. The preanesthesia clinic is the ideal setting for early evaluation of high-risk pregnant patients. Comorbidities may include cardiovascular pathology such as valvular abnormalities, septal defects, aortopathies, arrythmias and cardiomyopathies. Additional comorbidities include pulmonary conditions such as obstructive sleep apnea as well as preexisting neuromuscular and skeletal disorders that may impact anesthetic management. Hematologic conditions involving both bleeding diathesis and thrombophilias may present unique challenges for the anesthesiologist. Patients may also present with endocrinopathies including diabetes and obesity. While not as common, high-risk patients may also have preexisting gastrointestinal conditions such as liver dysfunction, renal failure, and even post-transplant status. Ongoing and prior substance abuse, obstetric conditions such as placenta accreta spectrum disorders, and fetal conditions needing ex utero Intrapartum treatment also require advanced planning. Preanesthesia evaluations also help address important ethical and cultural considerations. Counseling patients regarding anesthetic considerations as well as addressing concerns will play a role in reducing racial and ethnic disparities. Anticipatory guidance by means of pre-anesthetic planning can facilitate multidisciplinary communication and planning. This can allow for an impactful and meaningful role in the care provided, allowing for safe maternal care and optimal outcomes.

## Introduction and background

While caring for pregnant patients, current anesthesia guidelines emphasize the importance of a communication system between obstetric providers, anesthesiologists, and members of multidisciplinary teams [1]. Women are now entering pregnancy with significant physical and mental health disorders, and complex social challenges. The additional physiological burdens of pregnancy and delivery can be considerable for both the mother and the fetus and may convert a stable and well-controlled systemic condition into a deteriorating or even fatal one [2]. 

Early evaluation of high-risk pregnant women in preanesthetic clinics can enable optimal care. Preanesthetic clinics facilitate comprehensive patient assessment and optimization and provide education regarding obstetric management and anesthetic options for labor and delivery [3]. The American College of Obstetrics and Gynecology (ACOG) has generated recommendations for patients with concurrent comorbid conditions that generally call for anesthesiology consultation during pregnancy [4]. This consultation, in most cases, should take place near the end of the second trimester or early third trimester. Timing may be individualized depending on factors including severity of the condition and antepartum access to care. The medical, obstetric, and anesthetic plan for all high-risk patients must be communicated in advance to all providers who may cover the obstetric suite, with ability for 24-hour access to this information. In this narrative, we review systemic conditions that contribute to increased morbidity during pregnancy, including an increased risk for neuraxial or general anesthesia-related morbidity.

## Review

Cardiovascular system

The physiological changes of pregnancy can aggravate preexisting cardiac disease. Cardiovascular disease is a leading cause of maternal mortality. The severity of the maternal condition should not be underestimated and attributed to the “normal” symptoms of pregnancy [2]. A multidisciplinary team often follows these patients during pregnancy. Cardiac investigations recommended may include echocardiogram, electrocardiogram, magnetic resonance imaging and even cardiac catheterization. 

Patients with cardiac disease typically fall into the following broad categories-congenital versus acquired, valvular (stenotic versus regurgitant), septal or shunt lesions, pulmonary hypertension, ischemic heart disease, cardiomyopathies, aortopathies and arrythmias. Risk stratification models may be used to determine the best hospital for antenatal management and delivery [33,34]. For many low-risk patients with issues such as septal defects, mild to moderate regurgitant lesions, or corrected congenital lesions, where the actual medical, anesthetic, or obstetric issues are rarely problematic, a function of the antenatal anesthesiology consultation is to reassure patients that their risk is low [35]. 

Improvements in cardiac surgery have resulted in female babies with previously fatal congenital conditions surviving to reach sexual maturity [2]. Acquired cardiac disease may be attributed to valvular lesions in women from countries where rheumatic heart disease is more common, and to ischemic heart disease resulting from increased obesity, older maternal age, and smoking [36]. 

The effects of valvular disease in pregnancy vary based on several factors including type of lesion and severity. Ventricular dysfunction due to regurgitant valves can lead to heart failure and arrhythmias. Aortic regurgitation during pregnancy can be tolerated in the absence of left ventricular dysfunction. Stenotic valves are typically more concerning and when combined with the decreased systemic vascular resistance and increased blood volume in pregnancy, it can lead to syncope and congestive heart failure respectively. Echocardiographic measurement of valve areas is usually preferred to flow gradients during assessment since the latter can be expected to increase in pregnancy because of increased cardiac output and may not give as accurate an assessment (though a failure to increase may also suggest developing cardiac failure) [36]. Mitral stenosis is of the more common valvular defects seen in pregnancy. It can be associated with atrial fibrillation with risk of embolic episodes. Pulmonary hypertension may subsequently develop. Women with moderate to severe aortic stenosis may be at increased risk for pulmonary edema, arrhythmias, angina, and transient ischemic attacks or strokes. Pregnant patients may have undergone previous valve replacement. Mechanical valves require prolonged anticoagulation. The timing of discontinuation of anticoagulation has implications for safely performing neuraxial anesthesia.

Mortality due to pulmonary hypertension remains high during pregnancy despite advanced therapies. Vaginal delivery under appropriate analgesia is generally recommended. To avoid a rapid decrease in systemic vascular resistance that can cause cardiopulmonary decompensation, incremental epidural-only anesthesia with careful titration of local anesthetics is suggested, as general anesthesia with positive-pressure ventilation could increase pulmonary vascular resistance and right-to-left shunt [37]. General anesthesia should also be considered in patients under anticoagulation therapy or with heart failure who require mechanical ventilation.

Hypertrophic obstructive cardiomyopathy is frequently inherited and presents with left ventricular outflow obstruction. Tachycardias, hypovolemia and vasodilatation are poorly tolerated. Peripartum cardiomyopathy is the development of heart failure in the last month of pregnancy or within five months of delivery [38]. A small subset of patients demonstrate signs/develop symptoms during the second or third trimesters. Other causes of heart failure should be excluded and the absence of other recognizable cardiac disease before the last month of pregnancy should be verified. Dilated cardiomyopathy can also present in the peripartum period. Risk factors include previous history of peripartum cardiomyopathy and advanced maternal age.

For patients with syndromic (e.g. Marfan’s syndrome) and non-syndromic (e.g. ACTA2 gene mutations) familial aortopathies, monitoring blood pressure, symptomatology and surveillance imaging should be done throughout pregnancy to reduce risk of aortic dissections [33,39]. For parturients with history of arrhythmias due to conditions such as Wolf-Parkinson-White syndrome, anesthetic goals include avoidance of tachycardia. Beta-blockers reduce hemodynamic stress and are recommended during pregnancy. Additionally, in patients with pacemakers or automated implantable cardioverter defibrillators, the preanesthetic evaluation should include the reason for placement, information regarding type and functioning of device, battery life and time of last interrogation, patient dependence and response to magnet application all of which is facilitated by evaluation by the cardiac electrophysiology service [40].

Creation of a delivery plan, discussion of anesthetic and analgesic options, and discussion regarding invasive hemodynamic monitoring are part of the preanesthetic evaluation. Patients with severe left-sided obstructive heart disease including coarctation of the aorta and stenotic valvular lesions, pulmonary hypertension, and cardiomyopathies may have particularly poor outcomes and difficulty tolerating labor, surgery, and anesthesia [33-35]. High-risk patients may also benefit from the involvement of an experienced cardiac anesthesiologist in their peripartum care. Careful monitoring for clinical decompensation during delivery and in the first 72 hours postpartum should be anticipated. 

For most cardiac patients, vaginal delivery remains the safest option, barring obstetric indications for cesarean delivery. Vaginal delivery is associated with decreased blood loss and hemodynamic changes and reduced risk of thrombosis and infection [33]. A “cardiac vaginal delivery” may be recommended which involves little to no maternal pushing thereby avoiding potential hemodynamic compromise [34]. Planned cesarean section may be preferred in the highest risk patients, as it avoids the need for emergent delivery and permits the attendance of all relevant consultants [35]. Patients in this category include those in decompensated heart failure, severe pulmonary hypertension, severe left-sided obstructive lesions and some aortopathies. 

Anxiety and pain during labor can lead to increased afterload, decreased uteroplacental perfusion, and potential cardiovascular collapse. Neuraxial labor analgesia results in greater cardiopulmonary and hemodynamic stability and should be initiated as early as possible during labor.

As mentioned, parturients with cardiac disease may require invasive monitoring that includes placement of arterial, central venous and pulmonary artery catheters and transthoracic echocardiography. Central venous access allows for infusion of vasoactive drugs [35]. Pulmonary artery catheters are rarely used because of a lack of evidence of benefit in most situations coupled with serious risk of complications such as arrythmias, bleeding and risk of pulmonary artery rupture. Placement may be reserved for situations such as cesarean delivery in parturients with high-risk cardiac lesions, presence of pulmonary hypertension and where determination of cardiac filling pressures and cardiac output may be of immediate benefit [35].

Respiratory system

Pregnant women with obstructive (asthma, cystic fibrosis, bronchiectasis) or restrictive (fibrosing alveolitis, sarcoidosis, fibrosis) lung diseases may be seen in the preanesthetic clinic [36]. Asthma is the most common respiratory disease that accompanies pregnancy [41]. Adequate asthma control is assessed based on the frequency of symptoms and their severity, use of rescue therapy, history of exacerbations requiring systemic corticosteroid administration and results of pulmonary function tests. Education of patients is essential as exacerbations during pregnancy are often associated with non-adherence to treatment regimens. During the peripartum period, the asthmatic parturient should receive adequate hydration and analgesia as lack of pain control can trigger bronchospasm [37]. General anesthesia may cause exaggerated airway responses.

The physiological changes of pregnancy worsen sleep-disordered breathing, a spectrum of conditions of increasing severity from loud snoring to obstructive sleep apnea (OSA). All obstetric patients should be assessed for OSA as it increases the risk of adverse maternal, fetal, and neonatal outcomes [42]. If OSA is suspected, patients should be referred to a sleep medicine physician for evaluation and treatment. In cases of nonadherence, re-establishment of treatment should be encouraged and supported.

Cystic fibrosis patients begin pregnancy with mixed obstructive and restrictive lung disease, often with chronic lower respiratory tract infections [43]. Pulmonary function deteriorates as pregnancy progresses due to increased oxygen consumption, thicker mucous secretions and altered lung mechanics due to the enlarging uterus. Cystic fibrosis-related diabetes and intravenous access problems are common. Optimization includes increasingly intense daily physiotherapy. Neuraxial anesthesia is the method of choice for cesarean delivery, taking care to avoid excessively high blocks with risk of respiratory impairment [44]. Patients often prefer a semi-sitting position, which may make achievement of an adequate sensory block challenging. General anesthesia is avoided due to its propensity to worsen lung function. Postoperative care should be carried out in a critical care setting that can provide non-invasive and invasive ventilation if necessary.

Restrictive lung disease can occur secondary to intrinsic (e.g. sarcoidosis) or extrinsic (e.g. kyphoscoliosis) factors. Antenatal assessment should also include determination of respiratory reserve and functional status including exercise capacity testing and home oxygen requirements [44]. 

Neurological, neuromuscular and skeletal disorders

Pregnant patients with neurological disorders (e.g., epilepsy, multiple sclerosis, pseudotumor cerebri, Arnold-Chiari malformations, stroke, spinal cord injury, infections, tumors) neuromuscular disorders (e.g., myasthenia gravis) or musculoskeletal disorders (e.g., scoliosis, kyphoscoliosis, spinal dysraphisms, achondroplasia, chronic inflammatory arthritides) may also be encountered. Early consultation allows accurate documentation of any pre-existing neurological deficit and management. Delivery should be done in a tertiary care center with access to services such as neurology, neurosurgery, and radiology. 

Pregnant women with epilepsy face several challenges. Increased seizure frequency is observed in some due to the pharmacokinetic and hormonal changes of pregnancy. Anticonvulsant drug levels can decrease during pregnancy which is explained by decreased plasma protein binding and the greater drug clearance [45]. Maternal seizures can have a devastating effect on the fetus. Optimizing antiepileptic therapy before and during pregnancy is critical to maintaining prepregnant serum antiepileptic drug levels. All anti-epileptic drugs cross the placenta, and most are associated with congenital anomalies. Substituting new antiepileptic drugs after conception is not recommended due to the increased risk of teratogenicity associated with polytherapy [45]. The importance of continuing the anti-epileptic drug regimen throughout pregnancy must be reiterated. There is no contraindication to regional anesthetic blockade in epileptic parturients. 

Multiple sclerosis is a chronic autoimmune inflammatory demyelinating disease with an onset commonly occurring during the childbearing years. A lower relapse rate is thought to occur during pregnancy, with an increased relapse rate in the first three months postpartum. Patients should be counseled about this risk that exists irrespective of the type of anesthetic [45]. Assessment should include patient’s overall functional, pattern of deficits and systemic involvement. There is no contraindication to use of neuraxial anesthetic techniques for labor analgesia or surgery. Epidural anesthesia with low concentrations of local anesthetic may be preferred to avoid toxicity secondary to exposure of the demyelinated fibers to high concentrations of local anesthetic, as with spinal anesthesia. General anesthesia is also considered safe [46]. Core temperature should be monitored in the peripartum period as acute temperature changes can precipitate acute relapse [45].

Arnold-Chiari malformations (ACM) are congenital anomalies in which the cerebellum herniates through the foramen magnum, displacing the lower pons and medulla. Of the four types described, type I is the most common. The condition is associated with an impaired flow of cerebrospinal fluid (CSF) from the fourth ventricle and dynamic or static herniation of brain tissue. It has been shown that cerebrospinal fluid pressure increases with uterine contractions by a mean of 2.5 mmHg, and that the elevation of intracranial pressure (ICP) is much greater during the second stage of labor. While neuraxial techniques have been found to be a safe and viable anesthetic option, correct interpretation of imaging tests and multidisciplinary input, including that of neurology and neurosurgery, should be elicited before deciding on anesthetic management in patients with known increases in ICP [47,48].

Women with cerebral aneurysms or intracranial space-occupying lesions (e.g., tumor, hematoma, edema) present unique peripartum challenges. For patients with unruptured cerebral aneurysms, while mode of delivery does not seem to alter the incidence of rupture, most women tend to undergo cesarean delivery [49]. Epidural anesthesia has been performed in these patients, due to the theoretical concern that leakage of cerebrospinal fluid with spinal anesthesia may predispose to rupture [50]. For patients with a space-occupying lesion, neuraxial and general anesthesia pose additional considerations. Parturients with space-occupying lesions that have no mass effect, hydrocephalus, or clinical or imaging findings suggestive of increased intracranial pressure are likely to have minimal to no increased risk of herniation from a dural puncture. Parturients at high risk of herniation from a dural puncture are those with lesions that compress normal brain tissue causing it to shift across the midline or downward, with or without obstruction to the flow of cerebrospinal fluid. If they exhibit neurological changes from their baseline status or a rapidly progression lesion, neuraxial anesthesia may be contraindicated. Preparations should then be made for an intracranial pressure-controlled general anesthetic for surgery in these patients [51]. The preanesthetic evaluation may include referral for magnetic resonance imaging, neurosurgical consultation, and discussion of alternate anesthetic plans when neuraxial anesthesia is contraindicated [52,53].

Idiopathic or benign intracranial hypertension (also known as pseudotumor cerebri) is a common condition, characterized by raised intracranial pressure without related pathology in either the brain or the composition of cerebrospinal fluid. The main symptom is headache, and the cardinal sign is papilledema [54]. Treatment in severe cases often includes acetazolamide after 20 weeks’ gestation, short-term corticosteroids and possibly shunt placement or surgical management. The main goal of management is preservation of visual function. Despite the raised intracranial pressure in these patients, there are no specific contraindications to neuraxial techniques. Slow, incremental dosing of epidural medication may be better tolerated in symptomatic patients [54].

Spinal dysraphisms are neural tube closure birth defects that may be open or closed and are commonly seen in the lumbosacral area. In patients with surgically corrected open spinal dysraphisms, the terminal portion of the spinal cord typically lies at a vertebral level lower than normal. Dysraphisms may be inherently associated with the tethered cord syndrome. Patients often develop minor sensory, motor, and functional deficits of the lower limbs, bowel and bladder. Closed defects may or may not be associated with cutaneous stigmata. A tethered spinal cord has implications for neuraxial anesthesia with increased risk due to its association with a low-lying spinal cord, need for imaging, and often abnormal preanesthetic neurological status [55]. If spinal dysraphism is suspected but no imaging studies are available, it may be prudent to avoid neuraxial anesthesia.

With improvements in management and rehabilitation, more women with spinal cord injury are conceiving children. Women with spinal cord injuries may give birth vaginally. Patients with spinal cord injury at the level of T6 or above are at risk of developing autonomic hyperreflexia. Although pain perception is impaired in these women at or above T10, neuraxial anesthesia is the treatment of choice for pain arising from the pelvic organs. Early neuraxial labor analgesia is advisable. Continuous intraarterial blood pressure monitoring should also be considered during the peripartum period [56]. 

Myasthenia gravis is an autoimmune disorder characterized by episodes of skeletal muscle weakness that are made worse by activity. The course of disease during pregnancy is highly variable. The maternal physiological changes of pregnancy may require adjustments in the doses of anticholinesterase drugs. Patients may experience progressive respiratory compromise secondary to diaphragmatic elevation during pregnancy. Preanesthetic evaluation should assess the extent of bulbar and respiratory involvement and overall baseline muscle strength. Pulmonary function testing should be done if there is respiratory compromise. Since the uterus consists of smooth muscle, the disorder generally does not affect the first stage of labor. The second stage of labor requires the use of striated muscle, so an assisted vaginal delivery may be required [45]. Early neuraxial labor analgesia is also recommended. In patients with severe bulbar or respiratory compromise, general anesthesia may be required for operative deliveries.

Rare neuromusculoskeletal disorders that have multisystem manifestations include myotonic dystrophies, neurocutaneous syndromes like neurofibromatosis and tuberous sclerosis, and Guillain-Barre syndrome. In muscular dystrophies, cardiorespiratory involvement may be varied. Neuraxial techniques are preferred for labor analgesia and cesarean delivery. Severe disease may result in both airway and spinal abnormalities presenting challenges [45]. In neurofibromatosis, pregnancy may exacerbate the disease by increasing tumor growth. The patient’s current symptoms and known lesions should be assessed. Neck, laryngeal, neuraxial, intracranial and peripheral nerve tumors may occur and should be ruled out by appropriate clinical and radiological evaluation [45]. Epidural and spinal techniques have been described in patients with Guillain-Barre syndrome. A theoretical risk for adverse neurological events after neuraxial techniques may exist secondary to anesthetic toxicity or immunologic modulation. 

Musculoskeletal disorders including the inflammatory arthritides rheumatoid arthritis and ankylosing spondylitis can affect perioperative planning for the obstetric patient. Systemic manifestation involving the cardiovascular and respiratory system should be ruled out. Neuraxial anesthesia may also be technically challenging do to calcified interspinous ligaments and osteophyte formation limiting the patient’s ability to flex forward. Unexpectedly high blocks have been reported [57]. Challenges in positioning the parturient for delivery may be anticipated.

Scoliosis is a lateral deviation in the vertical axis of the spine with severity being determined by measurement of the Cobb angle. Most cases of scoliosis are idiopathic; however, some are associated with neuromuscular conditions such as myotonic and muscular dystrophies, Marfan’s syndrome, rheumatoid disease, osteogenesis imperfecta and achondroplasia. Pregnancy exacerbates the severity of the spinal curvature and cardiopulmonary abnormalities including restrictive lung disease and pulmonary vascular resistance. Neuraxial anesthesia may be offered, however it may be technically difficult with increased possibility of complications such as aberrant local anesthetic spread. Patients with achondroplasia are often delivered by cesarean delivery due to an inadequate maternal pelvis outlet with a normal-sized fetus. The small stature and spinal stenosis in these patients necessitate a reduction in the dose of local anesthetic required, therefore an epidural or combined spinal-epidural anesthesia may allow for better titration of the local anesthetic dose [55].

Parturients who have had prior back surgeries may also have scar tissue or removal of the ligamentum flavum that could pose an anatomic challenge for epidural placement and for anesthetic administration [58]. If a patient has adhesions from a previous surgery, local anesthetic may not spread uniformly, resulting in inadequate analgesia. Anesthesiologists may have better success with review of prior imaging, surgical history as well as ultrasonography-guided neuraxial placement.

Hematological disorders

Common causes of thrombocytopenia during pregnancy include gestational thrombocytopenia, autoimmune (previously called idiopathic or immune) thrombocytopenic purpura, and hypertensive disorders of pregnancy. Gestational thrombocytopenia is a benign self-limiting condition and accounts for most of the thrombocytopenia seen during pregnancy [59]. In most women, the platelet count is ≥100,000/microL with no increased bleeding or bruising. There is an increased incidence of autoimmune thrombocytopenic purpura diagnosed during pregnancy. Consultation with a hematologist is often recommended for treatment options. Treatment includes intravenous immunoglobulins and steroids. Treatment with steroids may also have the added advantage of increasing the platelet count to an acceptable range for neuraxial anesthesia.

If thrombocytopenia is discovered during pregnancy, an assessment of bleeding history and etiology is necessary. The risk of spinal epidural hematoma associated with a platelet count ≥70,000 × 10^6^/L is likely to be very low in obstetric patients with thrombocytopenia secondary to gestational thrombocytopenia, immune thrombocytopenia (ITP), and hypertensive disorders of pregnancy in the absence of other risk factors [60]. For patients with extreme or symptomatic thrombocytopenia or other refusal of blood products, alternative analgesia should be planned. Controlled inhalation of nitrous oxide or patient-controlled analgesia, with remifentanil, for example, can be used in patients who are not candidates for neuraxial anesthesia [61].

Congenital coagulopathies include von Willebrand’s disease which is divided into several subtypes based on quantitative and qualitative defects in the von Willebrand factor (vWF), which binds to collagen at sites of vascular injury, mediates platelet adhesion and aggregation, and serves as a carrier protein for coagulation factor VIII. A hematology consultation should be obtained early in pregnancy. These parturients should be tested for functional VWF and factor VIII concentration, as low levels are often be treated prior to delivery with therapies including desmopressin and von Willebrand factor concentrates. Case series have demonstrated safe spinal, epidural, and combined spinal-epidural anesthesia, and neuraxial anesthesia is considered an option for patients with normalized VWF:RCo, factor VII, and VWF antigen concentrations [61].

Common thrombophilias diagnosed in pregnancy include Factor V Leiden deficiency and protein C and S deficiency. ACOG has recommended thromboprophylaxis for pregnancies complicated by inherited thrombophilias, which present challenges for neuraxial anesthesia about which the patient should be counseled [62]. The Society of Obstetric Anesthesia and Perinatology recommendations for anticoagulation and safe neuraxial placement should be closely followed [13]. Patients with pre-existing lupus anticoagulant are at risk of thromboembolic events and many also require thromboprophylaxis.

Endocrine disorders

Gestational diabetes accounts for the majority of women with diabetes, followed by type I diabetes and type 2 diabetes [63]. Maternal and perinatal outcomes improve when maternal glycemic control approaches that in normal pregnancies. Preexisting diabetes can be associated with macrovascular and microvascular complications. Patient education and strict glycemic control with regular monitoring of capillary glucose by the patient is essential. A review of patient’s glycemic control, medications or insulin therapy is essential. Expected changes in insulin requirement at the time of delivery should be discussed with the patient [64]. Maternal insulin requirements decrease with the onset of labor, increase again during the second stage of labor, and decrease markedly during the early postpartum period. Intravenous glucose and insulin infusions during the peripartum period should be titrated to maintain tight maternal blood glucose control [64]. For patients with planned cesarean section, adjustment of the home insulin regimen would be needed to avoid hypoglycemia. Autonomic cardiovascular dysfunction and gastroparesis are additional anesthetic considerations.

Patients with obesity are at a higher risk of anesthetic and obstetric complications and should have a formal preanesthesia evaluation [65]. A thorough medical history should be obtained including screening for pulmonary and cardiac comorbidities. A comprehensive physical examination including airway, thoracolumbar area and sites for venous access is essential. Patients should be counseled regarding a higher incidence of difficult labor, failed labor, cesarean delivery and postpartum hemorrhage. The patient should be explained regarding anesthetic options for labor analgesia and cesarean delivery, and that neuraxial anesthesia placement may be challenging [66]. Patients should be educated that placement during early labor is optimal to allow sufficient time for placement and confirmation of block efficacy and reliability, to decrease the risk of neuraxial anesthesia failure and needing general anesthesia for an emergency cesarean delivery [66,67]. Neuraxial options include epidural, combined spinal-epidural, dural puncture epidural or continuous spinal techniques. Obesity may have a protective effect against the development of postdural puncture headache. Ultrasonography can reduce procedural times and placement failures for neuraxial anesthesia [67]. Due to technical challenges when using a Pfannenstiel incision in patients with a large pannus, surgeons may occasionally perform a cesarean delivery using a supraumbilical vertical midline incision, for which use of the double neuraxial catheter anesthetic technique has been described to obtain adequate anesthetic coverage [67]. Surgical plan for type of incision and position of the panniculus should be confirmed preoperatively. Availability of non-invasive ventilation, difficult airway equipment, specialized bariatric equipment and ultrasonography is part of preanesthetic planning for these cases [68].

Pregnant women likely exhibit overt or symptomatic hypothyroidism at a much lower rate than nonpregnant women. However, the required replacement of thyroid hormone in clinically hypothyroid patients often increases during pregnancy and is titrated to serum TSH levels. Patients with hyperthyroidism are treated with methimazole and propylthiouracil. Radioactive iodine is contraindicated in pregnancy. Patients with severe, untreated hyper- or hypothyroidism may present with unexplained tachycardia (hyperthyroidism) or bradycardia (hypothyroidism), abnormal weight gain for gestational age, or a visible or palpable goiter [64]. This can lead to airway and hemodynamic challenges for the anesthesiologist. These patients may also need additional consultation from an endocrinologist as well as coordination for either medical management or surgery. If a patient presents with signs of an impending thyroid storm, they may need an emergency cesarean section and close medical management including the use of corticosteroids and beta-blockers.

Gastrointestinal disorders

Pregnant patients with chronic liver disease or end-stage liver disease who are pre-transplant are at increased risk for both obstetric and anesthesia-related complications [69]. They are at a high risk for obstetric hemorrhage, paradoxical thromboembolic events, and are predisposed to development of pulmonary hypertension or cardiomyopathy [70]. Post-orthotopic liver transplant patients will benefit from screening for transplant-related complications, graft function, persistent symptoms and immunosuppression-related comorbidities. Thrombocytopenia may persist. Neuraxial anesthesia may be acceptable if their coagulation profile and platelet count is within normal limits [70].

Parturients with chronic kidney disease or end-stage renal disease are anemic, experience hemodynamic alterations and electrolyte imbalances and are at increased risk for gastric aspiration. Although uncommon, pregnancy has been reported in women undergoing hemodialysis [71]. Anticoagulation with heparin, thrombocytopenia and platelet dysfunction pose an increased bleeding risk during delivery and may preclude neuraxial anesthesia in end-stage-renal disease patients [70] Patients who are receiving adequate dialysis are less likely to have significant platelet dysfunction and their risk of bleeding is less [72]. Patients may also be hyper- or hypovolemic at the time of delivery due to the timing of dialysis.

Substance abuse disorders

Tobacco and alcohol are among the most commonly used substances during pregnancy and can cause anesthetic complications in addition to their teratogenic effects [73]. Anesthesiologists can play a vital role in smoking cessation. The “Ask-Advise-Refer” approach involves asking patients about their smoking history, advising them to quit based on its harmful health effects, and referring them to “Quitline” services [74]. Intrauterine alcohol exposure is the leading cause of preventable birth defects in the United States [75]. Cessation of alcohol consumption must be encouraged during pregnancy.

There is a high prevalence of opioid use during pregnancy, with risks of maternal withdrawal, neonatal abstinence syndrome, and increased analgesic needs due to opioid intolerance or opioid-induced hyperalgesia [76]. Parturients with chronic opioid use may present in various stages of opioid addiction including current abstinence, medication-assisted treatment or an untreated use disorder. Questions regarding their substance abuse disorder should be asked in a respectful and non-judgmental manner [75]. Assessment should include developing a mutually agreeable strategy for pain management with appropriate goals for pain intensity scores. Patients receiving opioids regularly have fears that their labor pain will not be perceived, or pain medication will be withheld. Referral to appropriate addiction resources with education about the maternal, fetal, neonatal, and anesthetic complications caused by substance use should be done [73]. Chronic opioid requirements or pharmacological maintenance therapy should be continued during the intrapartum period, with a plan for multimodal therapy. Women in medication-assisted treatment programs are often managed with methadone or buprenorphine. The precise dose should be confirmed, and any dose adjustments must be done in consultation with the prescribing physicians. Opioid tolerant patients also have higher rates of comorbid psychiatric illness and may benefit from an additional psychiatric consultation [76,77]. Patients who are at risk for substance abuse should be evaluated for their current status, history of opioid use and recreational drug use, relapses, and triggers [77]. Marijuana use may enhance the risk of myocardial depression perioperatively with synergistic effects in combination with alcohol, benzodiazepines, cocaine, or amphetamines [75]. Cocaine and methamphetamine use in pregnancy by themselves can cause serious systemic complications [75]. Cocaine use is associated with increase in circulating catecholamines resulting in tachycardia and hypertension which may manifest as preeclampsia or even myocardial ischemia. Cocaine-induced thrombocytopenia may occur and even warrant a platelet count [78]. Concomitant use of cocaine can result in profound hypotension with neuraxial anesthesia and arrythmia with general anesthesia. Acute and chronic methamphetamine use can also cause perioperative hemodynamic instability and arrythmias.

High-risk obstetric and fetus-related considerations

Patients with placenta accreta spectrum disorders benefit from an early preanesthetic consultation. These cases involve multidisciplinary care and coordination involving maternal-fetal medicine, gynecology-oncology, obstetric anesthesiology, urology, general and trauma surgery, interventional radiology and neonatology, with potential need for massive transfusion [79]. The obstetrician will plan for a cesarean delivery and possible immediate or delayed hysterectomy . Discussion with the patient should include placement of large-bore peripheral or central venous access, arterial cannulation, and anesthetic techniques which may include neuraxial anesthesia, primary general anesthesia or a combined neuraxial-general anesthesia [79].

Ex utero intrapartum treatment is a specialized surgical technique developed to establish cardiopulmonary support safely and efficiently at delivery while maintaining placental bypass. The technique involves planned partial delivery of the fetus via hysterotomy while maintaining uterine relaxation and placental support, allowing for the establishment of neonatal cardiopulmonary stability in a controlled manner [80]. Early anesthesiology consultation is part of the multidisciplinary planning and coordination needed in these cases [80].

We have included the following tables for further guidance. Tables 1 and 2 summarize components of the obstetric preanesthetic assessment that have specific considerations [5-12]. Table 3 summarizes benefits, risks, and alternatives of neuraxial and general anesthesia when providing counseling to patients [4,13-15]. Table 4 addresses some common patient questions involving obstetric anesthesia [4,16-22]. Table 5 addresses important ethical and cultural obstetric anesthesia considerations [23-28]. Point-of-care ultrasonography is also increasingly being used in obstetric anesthesia and its applications are reviewed in Table 6 [29-32]. All these components present unique challenges to the anesthesiologist.

**Table 1:** Obstetric anesthesia consultation: history-taking.

System	‘Red Flags’ conditions
Neurological	Intracranial or spinal tumors or aneurysms, Arnold-Chiari malformations, multiple sclerosis, seizure disorders, radiculopathies, neurocutaneous syndromes Pre-existing neurological deficit, changes in baseline neurological status
Neuromuscular disease	Myotonic dystrophies, muscle dystrophies, myotonias, motor neuron disorders Myasthenia gravis, spinal cord injury
Cardiovascular	Surgically corrected or uncorrected congenital disease, significant valvular or regurgitant lesions, heart failure, pulmonary hypertension, inherited or acquired arrhythmogenic disorders, aortopathies, cardiomyopathies, coronary artery disease Pacemakers, automatic implantable cardioverter-defibrillators, ventricular assist devices
Respiratory	Obstructive disorders (poorly controlled asthma), restrictive disorders (sarcoidosis) Functional reserve and rate of disease progression Pandemic disease (Recent or prior COVID-19 infection requiring hospitalization)
Endocrine	Diabetes (peripartum insulin regimen and insulin pump management) Uncontrolled thyroid disorders, adrenocortical insufficiency Obesity and associated metabolic syndromes
Gastrointestinal	End stage renal disease, significant liver disease, prior abdominal or transplant surgery
Hematological and coagulation disorders	Idiopathic thrombocytopenic purpura, Von Willebrand disease, Congenital factor deficiencies, parturient on pharmacological thromboprophylaxis
Psychiatric disorders	Schizophrenia spectrum disorders Depression and suicidal risk Substance abuse(cocaine, amphetamines, heroin, prescription opioids) Opioid tolerance/withdrawal issues, pharmacological maintenance therapy with buprenorphine or methadone, chronic pain issues
Obstetric conditions	Risk factors for hemorrhage (multiple gestation, multiparity, placenta previa) Placenta accreta spectrum disorders ex-utero intrapartum treatment procedures
Miscellaneous	Syndromic disorders, congenital or acquired multisystemic disorders, malignant hyperthermia Language barriers, religious, ethical or cultural concerns, minors, fixed beliefs or myths regarding obstetric anesthetic care
Allergies	Mast cell activation syndrome, allergy to local anesthetics, latex allergy, severe food or medication allergies causing anaphylaxis

**Table 2:** Obstetric anesthesia consultation: physical examination.

Component	“Red Flags” on physical examination	Management and counseling
Body habitus	Obesity, Achondroplasia	Use point-of-care ultrasound. Epidural placement early in labor Expect difficulty with intravenous access and neuraxial placement
Airway	Difficult airway (Pregnancy by itself is a risk factor for a difficult airway) Increased Mallampati scores, small thyromental distance, large neck circumference, limited neck and jaw mobility, obesity Tongue, nose, lip piercings	Difficult airway equipment in the obstetric unit Encourage epidural placement early in labor Remove jewelry
Skin	Burn scars Bullae (epidermolysis bullosa), Hives (mast cell activation syndromes), Signs of intravenous drug use (e.g. injection marks) Markers for skin dysraphism & neurocutaneous syndromes (imaging to rule out tethered spinal cord or lesions)	Precautions for diagnostic and therapeutic interventions (needle electrodes for electrocardiogram, clip on pulse oximetry probes, silicone tape, padding for blood pressure cuff Encourage epidural placement early in labor to avoid stress-induced skin lesions Difficulty with intravenous access
Neuraxial	Previous back surgery (surgical scars) Obvious spinal deformity (scoliosis) Conditions such as Ehlers-Danlos syndromes associated with dural ectasia Infection (possible contraindication) Lumbar Tattoos (avoid if feasible)	Imaging to determine feasibility of neuraxial anesthesia Ultrasound for level of placement Neuraxial anesthesia may be more technically challenging with higher rate of failure
Implantable electronic medical devices	Cerebrospinal & lumboperitoneal shunts Pacemakers, automatic implantable cardioverter defibrillators, ventricular assist devices Vagus nerve stimulator therapy (bradycardia after epidural placement, laryngopharyngeal dysfunction Spinal Cord stimulators (neuraxial anesthesia is feasible, avoid wires) Insulin Pump (placement away from surgical area)	Understand principles of working and location to avoid device malfunction, device destruction, and ensure patient safety Ensure device is working Determine exact level of placement Understand interaction with electromagnetic fields (e.g. cautery) Understand interaction with monitoring

**Table 3:** Suggestions for patient counseling: neuraxial versus general anesthesia in obstetrics (benefits, risks and alternatives).

Neuraxial anesthesia	General anesthesia	Alternatives for labor analgesia
Benefits of Neuraxial Anesthesia:	Maternal and Fetal Risks of General Anesthesia:	Consider when Neuraxial Techniques are Contraindicated:
Increased maternal and paternal participation and satisfaction with the birth experience	Respiratory complications (Incidence of failed obstetric intubation about 1:300, aspiration, respiratory depression after emergence)	Nitrous oxide
Immediate post-delivery skin-to-skin bonding and breastfeeding	Avoidance of Cerebrovascular injury related to endotracheal intubation in women with co-morbidities (preeclampsia, cardiac disease)	Partial Opioid agonist-antagonists (Butorphanol, nalbuphine)
Risks of Neuraxial Anesthesia:	Uterine atony and predisposition to Obstetrical hemorrhage	Opioids (Morphine or fentanyl boluses, Fentanyl or remifentanil Patient controlled analgesia)
Post Dural puncture headache	Surgical site infections	Ketamine
Neurological injury	Postpartum depression	
Failed spinal or epidural anesthetic, High neuraxial block	Inability to provide neuraxial opioids and increased pain after cesarean delivery	
Epidural abscess/ meningitis	Venous thromboembolic complications	
Epidural hematoma	In utero exposure to anesthetic drugs with potential neurobehavioral effects	
	Reduced Apgar Scores	

**Table 4:** Neuraxial labor analgesia: questions, myths and suggested answers based on current literature.

Do epidurals prolong labor?	Do epidurals cause chronic backache?	I would prefer to have a “natural” delivery (intravenous analgesics are often acceptable) without an epidural.	Is there such a thing as “too early” or “too late” to get an epidural? I will not be able to “push” well and may need a cesarean delivery.	Do epidurals cause autism?
Epidurals prolong the second stage of labor by a mean difference of 7.66 minutes without negative effects to the fetus and neonate.	There is no relationship between labor epidural analgesia and long-term postpartum back pain.	Systemic opioids can be associated with adverse maternal and fetal effects.	In the absence of a medical contraindication, maternal request is a sufficient medical indication for pain relief during labor.	No association has been found between epidural labor analgesia exposure and an increased offspring risk of autism spectrum disorders.
A low concentration of epidural local anesthetic does not greatly affect the duration of the second stage of labor.	Chronic backache can result from unintended dural puncture.	Labor pain can be associated with adverse maternal and fetal effects all of which are obtunded with effective epidural analgesia.	Neuraxial analgesia does not appear to increase the cesarean delivery rate and therefore should not be withheld for that reason. There is also no increased risk of instrumented vaginal delivery with the lower concentration of local anesthetics used in modern epidurals.	
		Placement of an epidural catheter early during the course of labor is recommended to avoid risks of general anesthesia in the event of cesarean delivery.	There is also no difference in the rate of cesarean delivery with early (before 4-5 cm cervical dilation) versus late (after 4-5 cm cervical dilation) epidural analgesia.	

**Table 5:** Obstetric preanesthetic assessment: ethical and cultural considerations.

Patient considerations	Description of issues
Minors (In the Unites States, where the age of majority is set by individual states, minor usually refers to someone under the age of 18)	Pregnancy, in itself, does not grant legal emancipation, but often gives the minor authority to consent for medical procedures involving her pregnancy and baby. May not have full understanding of implications due to level of education and health literacy. Encourage involvement of family members or advocates during discussions
Racial and Ethnic Disparities	Minority women (African-American and Hispanic) less likely to use Neuraxial Labor Analgesia. Patient, provider, and systems-related causes are responsible. Anesthesiologists can address misconceptions and provide education using interpreters, thereby allaying anxiety and improving health literacy
Jehovah’s Witness	Refusal of allogeneic blood transfusion is a constitutionally protected right recognized by the US supreme court and statutory provisions. Parturient must be interviewed in a non-coercive environment and confidentiality must be maintained. Use of preoperative pharmacological therapy to increase hematopoiesis. Some cellular and plasma derivatives may be acceptable as “personal conscience” items and differ amongst witnesses. Discuss intraoperative cell salvage, acute normovolemic hemodilution and epidural blood patch with maintenance of a closed circuit continuous flow system. Discuss use of alternatives such as albumin, cryoprecipitate, and fibrinogen concentrate. Verification of acceptable treatments and accurate documentation in the medical record. Involvement of hospital liaison committees and risk management personnel if needed
Psychiatric Illness	Relapse of illness may occur during pregnancy due to discontinuation of medications secondary to concern for teratogenicity. Does not automatically mean that patient lacks capacity. Patient who lacks capacity during pregnancy may regain it during labor and delivery. Appreciating patient’s level of understanding and capacity for decision-making Involvement of legal and risk management if necessary

**Table 6:** Application of point-of-care ultrasound (POCUS) in obstetric anesthesia.

Exam	Examples of applications
Airway assessment	Predictors of difficult airway (measurement of hyomental distance with neck extended, thickness of anterior neck soft tissue)
Airway management	Diagnosis of bronchospasm and endobronchial intubation, identification of cricothyroid membrane (useful to mark in obese patients)
Optic Nerve	Optic nerve sheath diameter to a) Diagnose intracranial hypertension (useful in preeclampsia) b) Assessment of cerebrospinal fluid leak and to supplement epidural blood patch
Cardiac	Assessment of volume status, (preload), myocardial contractility, end-diastolic volumes of both ventricles (utility in preeclampsia and cardiomyopathies)
Pulmonary	Identification and diagnosis of interstitial fluid, pulmonary edema, pleural effusion, consolidation, atelectasis, pulmonary embolism, pneumothorax (Assessment of dyspnea, diagnosis of cause of hypoxia)
Vascular Access	Peripheral and central venous cannulation (minimize puncture attempts and arterial cannulation), arterial cannulation for invasive monitoring
Neuraxial	Identification of desired interspace, optimal puncture site (midline or paramedian), angle of needle insertion and trajectory, estimated depth to the epidural space (minimized failed and traumatic attempts, needle redirections and useful in obesity, scoliosis)
Abdomen	Truncal nerve blocks (Transversus abdominis plane or TAP blocks, Quadratus lumborum blocks) for post cesarean analgesia
Gastric	Quantitative and qualitative assessment of gastric contents (a clinical tool for assessment of aspiration risk, if the stomach is known to be empty, rapid sequence induction and associated inherent risks at intubation may be avoided)

## Conclusions

Anesthesiology consultations for complex obstetric patients foster optimal outcomes. They can help facilitate multidisciplinary care and enable shared decision-making. Furthermore, understanding the physiologic changes during pregnancy and the complex interplay between systemic comorbidities can improve the care provided. Finally, improved communication with patients regarding expectations, and anesthetic and peripartum planning can improve parturients’ satisfaction with their delivery experience and potentially reduce maternal morbidity and mortality.
